# Application of robotic transcranial Doppler for extended duration recording in moderate/severe traumatic brain injury: first experiences

**DOI:** 10.1186/s13089-018-0097-0

**Published:** 2018-07-23

**Authors:** F. A. Zeiler, P. Smielewski

**Affiliations:** 1 Division of Anaesthesia, Department of Medicine, Addenbrooke’s Hospital, University of Cambridge, Cambridge, UK; 2 Department of Surgery, Rady Faculty of Health Sciences, University of Manitoba, Winnipeg, Canada; 3 Clinician Investigator Program, Rady Faculty of Health Sciences, University of Manitoba, Winnipeg, Canada; 4 Section of Brain Physics, Division of Neurosurgery, Department of Clinical Neurosciences, Addenbrooke’s Hospital, University of Cambridge, Cambridge, UK

**Keywords:** Robotic transcranial Doppler, TCD, Traumatic brain injury, TBI, Technology review

To the Editor,

Long duration application of transcranial Doppler (TCD) for recording of middle cerebral artery (MCA) cerebral blood flow velocity (CBFV) has been fraught with difficulties [1, 2]. Classically, TCD has been labor-intensive, with limited ability to obtain uninterrupted recordings for extended periods. Furthermore, application of TCD within neurocritically ill for long durations has been limited given the complexity of care, regular bedside nursing care/patient manipulations, and presence of various other multi-modal monitoring devices. This is especially the case in traumatic brain injury (TBI) patients, with the adoption of extensive multi-modal monitoring. Within TBI, most TCD recordings, using standard widely available probes and holders, range from 30 min to 1-h duration and are frequently interrupted due to shifting of the probe and signal loss [3, 4]. Thus, we are typically left with a “snap-shot” recording with TCD examination, limiting our ability to extract valuable continuous variables, such as autoregulatory capacity [3–5].

Recent advances in robotics have led to the development of robotically driven TCD examination probes, integrated with automated algorithms for MCA CBFV detection and optimization of recorded signal intensity. To date, these devices have not been readily applied to the neurocritically ill, particularly moderate and severe traumatic brain injury (TBI) patients. However, given this advancement, they provide the potential to improve dramatically our ability to obtain longer, uninterrupted, TCD recordings in this population.

Within, we provide a review of our initial experience with the application of a new robotic TCD system, the Delica EMS 9D robotic TCD, in 10 moderate and severe TBI patients undergoing multi-modal invasive/non-invasive cranial monitoring. To our knowledge, this is the first account of the application of this device within the critically ill TBI population.

## Methods

From November 2017 to January 2018, in place of our regular TCD devices [Doppler Box (DWL Compumedics, Singen, Germany) or Neuroguard (Medasonic, Fremont, CA, USA)], we applied the Delica EMS 9D robotic TCD device (Delica EMS 9D System, Shenzen Delica Medical Equipment Co. Ltd, China; http://www.delicasz/com) system for bilateral middle cerebral artery CBFV recording in moderate and severe TBI patients within the neurosciences critical care unit (NCCU) at Addenbrooke’s Hospital, University of Cambridge. A total of 10 patients were recorded with this device during this time period. TCD monitoring is considered part of standard NCCU patient care. The timing to application of TCD-based monitoring varied from patient to patient, typically initiated between 24 h and 10 days post-TBI. We were only interested in applying the device in those TBI patients with concurrent extensive multi-modal monitoring (see list of devices below).

All 10 patients were intubated and sedated given the severity of their TBI, with ICP goals of less than 20 mmHg, and CPP goals of greater than 60 mmHg. Arterial blood pressure (ABP) was obtained through radial arterial lines connected to pressure transducers (Baxter Healthcare Corp. CardioVascular Group, Irvine, CA). All 10 patients had a frontally situated cranial bolt (Technicam Ltd, Newton Abbot, UK), for parenchymal ICP monitoring (Codman ICP MicroSensor; Codman & Shurtleff Inc., Raynham, MA), brain tissue oxygenation (Licox probe; Integra, Licox Brain Oxygen Monitoring System, Plainsboro, NJ), and cerebral microdialysis (M Dialysis AB, Stockholm, Sweden). Finally, bifrontal near infrared spectroscopy was also applied (NIRO-200, Hamamatsu Photonics Ltd, Japan).

We recorded all physiologic signals in digital high-frequency format (100 Hertz (Hz) or higher) using ICM+ software (Cambridge Enterprise Ltd, Cambridge, UK, http://icmplus.neurosurg.cam.ac.uk). This was installed and run directly off the Delica monitor. TCD was recorded at 100 Hz, while ICP and ABP were recorded at 124 Hz (i.e., the maximum frequency available from our NCCU General Electric (GE) Solar monitors). NIRS signals were up-sampled by ICM+ from their native set frequency of 0.5 Hz to match the frequency of the recorded TCD, ICP and ABP signals, allowing for a synchronized time series across all recorded modalities.

As the goal was to assess our first experiences with application of this new robotic TCD device within this particular TBI patient population, focusing on the advantages and disadvantages of the device, we will, therefore, not provide any further patient information or clinical outcomes. The following sections will describe the device and outline the advantages/disadvantages of the system within the moderate/severe TBI population. Finally, we will make a conclusion regarding the product.

## The device—an overview

### The probes/robotic drive

**Fig. 1:**
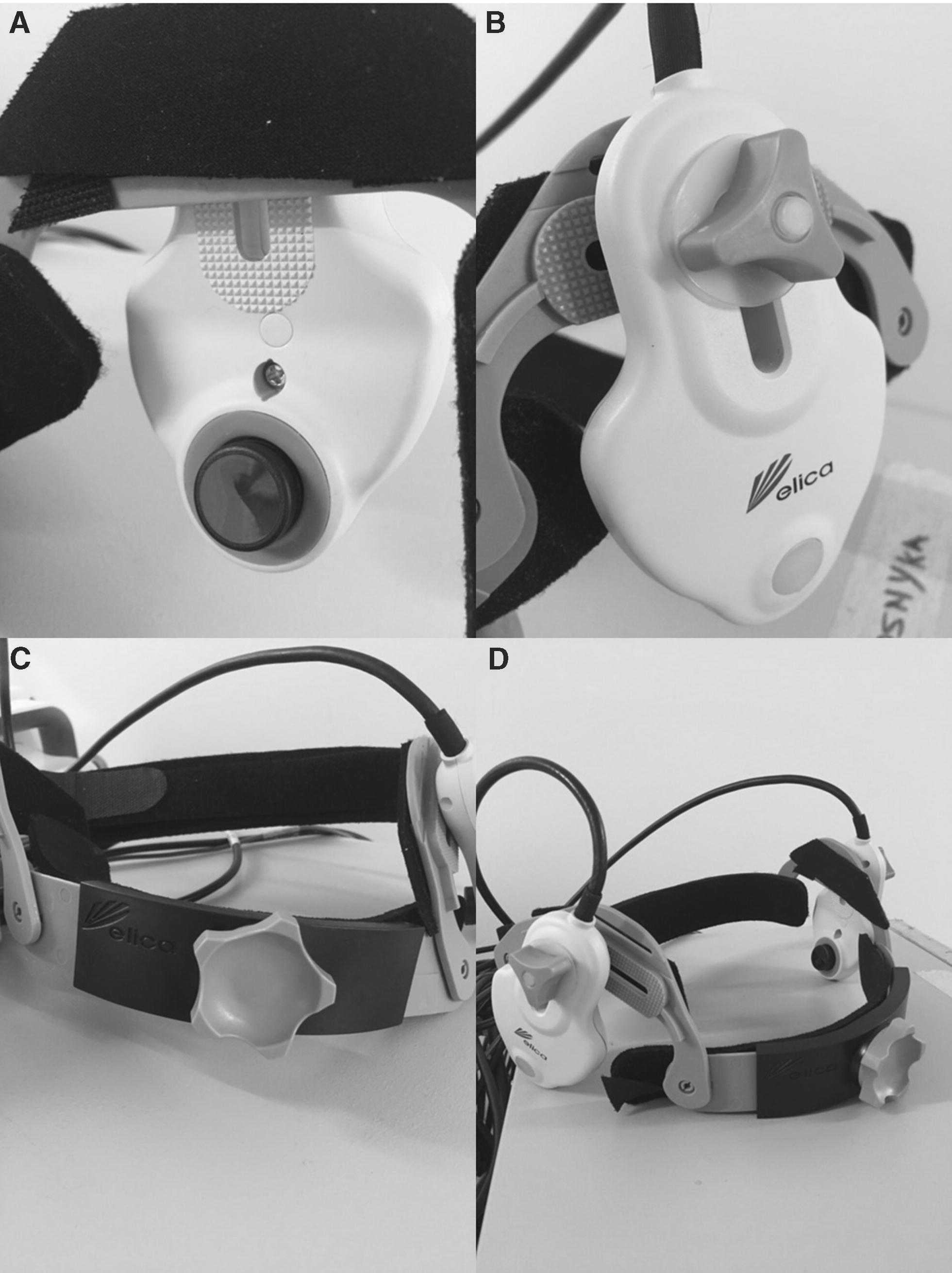
Delica EMS 9D robotic TCD probe and headband. *TCD* transcranial Doppler. **A** TCD probe (black circular object) held with robotic drive as one construct within plastic casing. Medial view—where probe contacts patient for transtemporal insonation of the middle cerebral artery. Neoprene pad is also seen on medial aspect of head-band. **B** Lateral view of robotic drive/TCD probe construct. Displays wing-nut attachment to head-band holder, via “inverted U-shaped” plastic bracket. **C** Anterior view of head-band displaying plastic component and wheel ratchet system for tightening. **D** Full view of head-band with bilateral robotic drive/TCD probes attached

The Delica EMS 9D robotic TCD system is a portable TCD system allowing for bilateral simultaneous MCA insonation. The standard probes available with the system are 1–2 MHz Doppler ultrasound probes, each attached to a separate robotic drive. The entire drive/probe construct is encased within a tough plastic shell and supported using a head-band type frame (Fig. 1A). An option exists for the probes are also surrounded by a small rubber ring around its periphery, which is designed to hold ultrasound gel for longer, allowing for preserved signal quality (See Additional file 1). Figure 1 displays various pictures of the Delica EMS 9D system.

The head-band frame is a composite of plastic and Velcro straps with fabric (Fig. 1C and 1D). The diameter of the head-band may be adjusted using either the Velcro straps, or the ratcheting wheel located on the front of the head-band (Fig. 1C). Ideal location of the band is just above the orbital margins. Near the temporal windows, the head-band frame contains plastic inverted “U-shaped” pieces following the course of the superior temporal line, meant for mounting the robotic drive/TCD probe construct (Fig. 1B). This is accomplished using wing-nut fasteners. The entire head-band is padded with exchangeable Neoprene inserts, for comfort (Fig. 1A).

### The monitor—gross interface

The Delica EMS 9D monitor is designed for portability, with a carrying handle situated at the top. The device itself is a Windows based machine, allowing for the installation of various other Windows based software packages directly onto the machine. As mentioned above, the device supports bilateral 1–2 MHz Doppler probes. However, it also has the ability to record signals with a single 4, 8, or 16 MHz probe. The device also supports a touch screen method of interaction with the software. Various ports are present on either side of the monitor, including: two external VGA monitor ports, four USB 2.0 ports, two USB 3.0 ports, one HMDI port, one serial RS232 COM port, and one ethernet port.

### The Delica TCD software

**Fig. 2:**
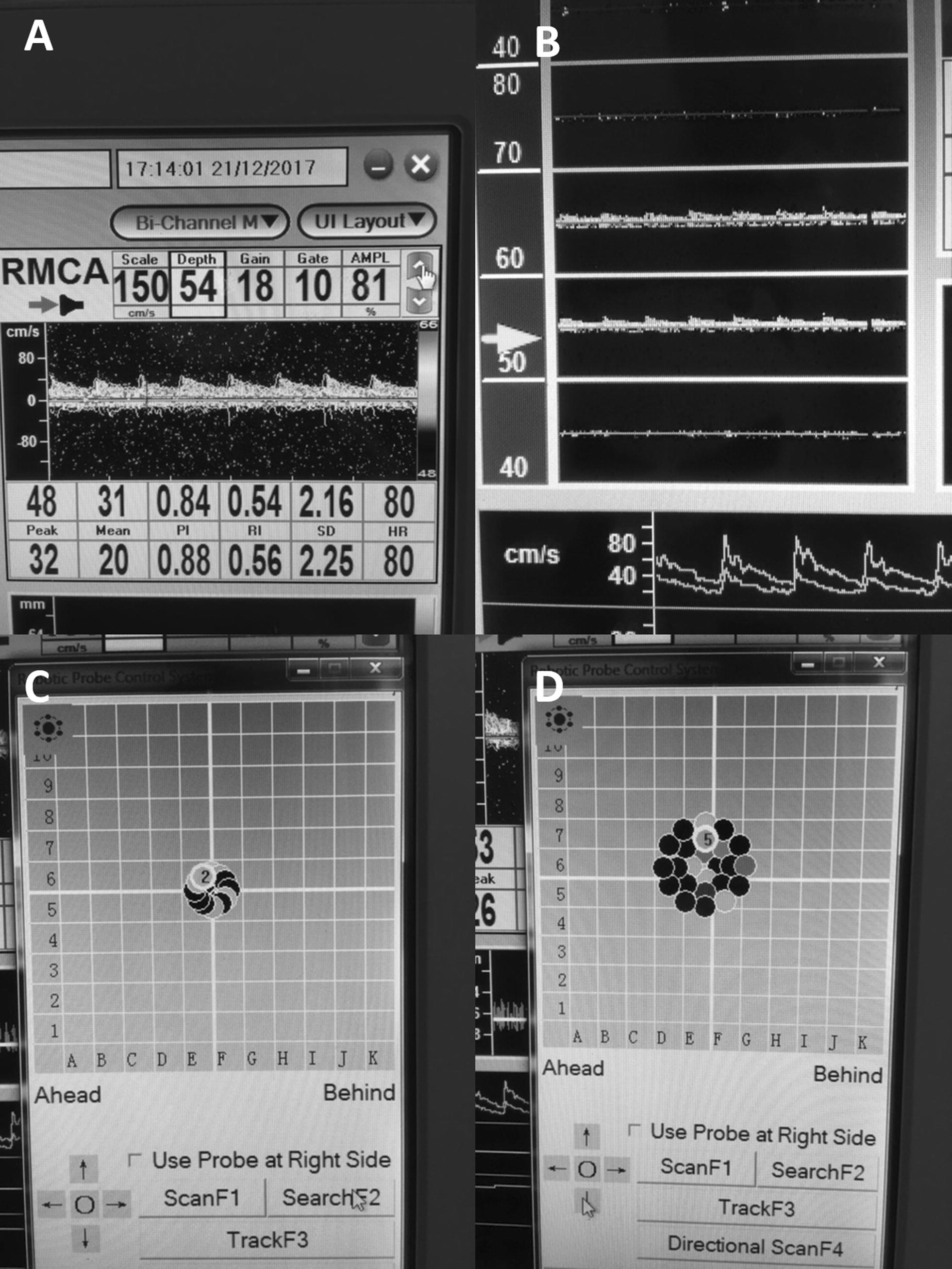
Delica EMS 9D TCD software interface. *TCD* transcranial Doppler. **A** Displays right middle cerebra artery real-time blood flow velocity waveform. **B** Displays sequential “look ahead” view of right middle cerebral artery, providing sequential real-time cerebral blood flow velocity waveforms at various depths of insonation (in this panel the presence/absence of flow velocity waveforms can be seen at depths of 45, 55, 65 and 75 mm in front of the probe face). **C** Search robotic signal acquisition function window, displaying the pattern at which the TCD probe is automatically moved. **D** Directional robotic signal acquisition function window, displaying the pattern which the TCD probe is moved, changing the angle of insonation. **C** and **D**, the various points of insonation are color coded to represent strength of signal, this can be appreciated via different shadings in this greyscale picture

The Delica EMS 9D comes with its own specially designed software for recording TCD signal. This provides both an easy to use interface with the robotic drive system, and continuously updating CBFV waveforms and M-mode signals. Furthermore, the left side of the screen provides both simultaneous left and right CBFV waveforms at various depths of insonation, allowing for selection of the optimal depth for recording. Various other more complex functions are available within the software, such as microemboli detection, however, we will not focus on these, given the goal was to assess the basic ability to record in critically ill TBI patients. Figure 2 Displays the software interface.

Finally, the robotic drive system carries four main functions: scan, search, direction, and track. Scan is an automated algorithm which moves the TCD probe position in a square grid pattern, insonating at each spot, assessing for MCA CBFV signal intensity and providing a color code for its findings (black = poor/no signal found, red = good; with colors ranging from blue, to green, to yellow, to orange, to red). It must be acknowledged that the amount the probe can change in position is limited, so large position corrections still required manual manipulation. After completion of the grid, the proprietary automated algorithm then chooses the best position. The search function provides an automated circular search pattern around the initial starting point, changing both probe position and insonation angle. As with the scan function, it insonates each spot, finding the optimal signal intensity for a final position. The directional function alters the TCD probe angle, using an automated algorithm, with the goal of finding the optimal insonation angle. It should be noted, at any point, the operator can manually change the probe position using the direction touch pad on the screen.

**Fig. 3:**
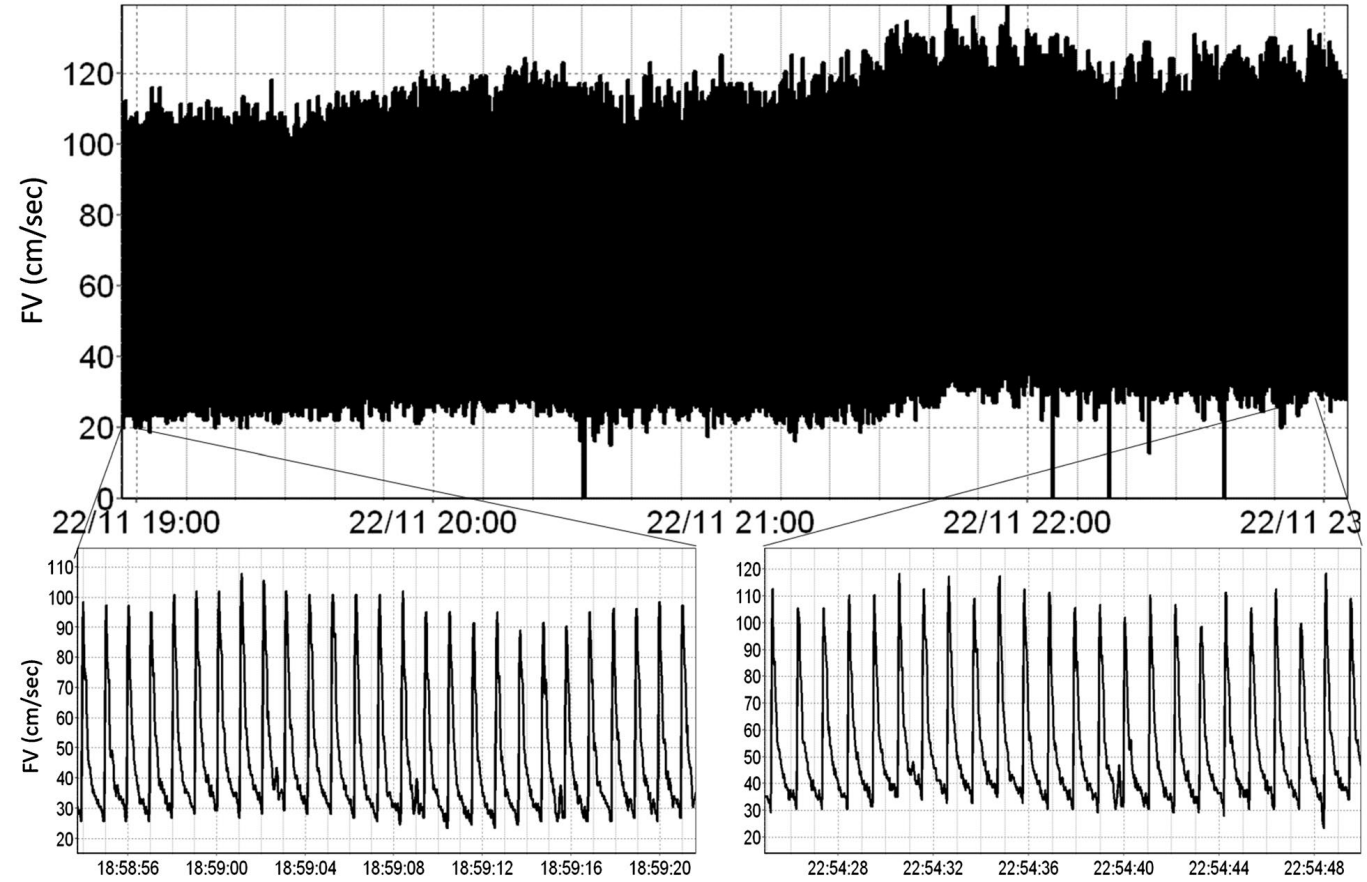
TCD CBFV signal—Over 4 h recording session. *CBFV* cerebral blood flow velocity, *cm* centimeter, *FV* flow velocity, *sec* seconds, *TCD* transcranial Doppler. *Diagram depicts continuous uninterrupted FV signal over 4+ h of recording. Two windows on bottom of image depict signal waveforms from beginning (bottom left) and end (bottom right) of the recording, demonstrating preserved high-quality signal throughout the duration of the lengthy recording period

Finally, the track function is enabled after the user selects the optimal position of insonation. This function is designed to automatically detect any deterioration in signal quality/intensity, and then automatically adjust the TCD probe (both via position and angle), using a proprietary algorithm, to restore optimal signal. Figure 3, demonstrates TCD CBFV signal recorded over a 4-h period, displaying continuous uninterrupted acquisition of maximum envelope flow velocity signal which retained good quality without the need of any manual adjustments over the whole duration of recording.

## Application in moderate/severe TBI—initial impression—advantages and disadvantages

**Fig. 4:**
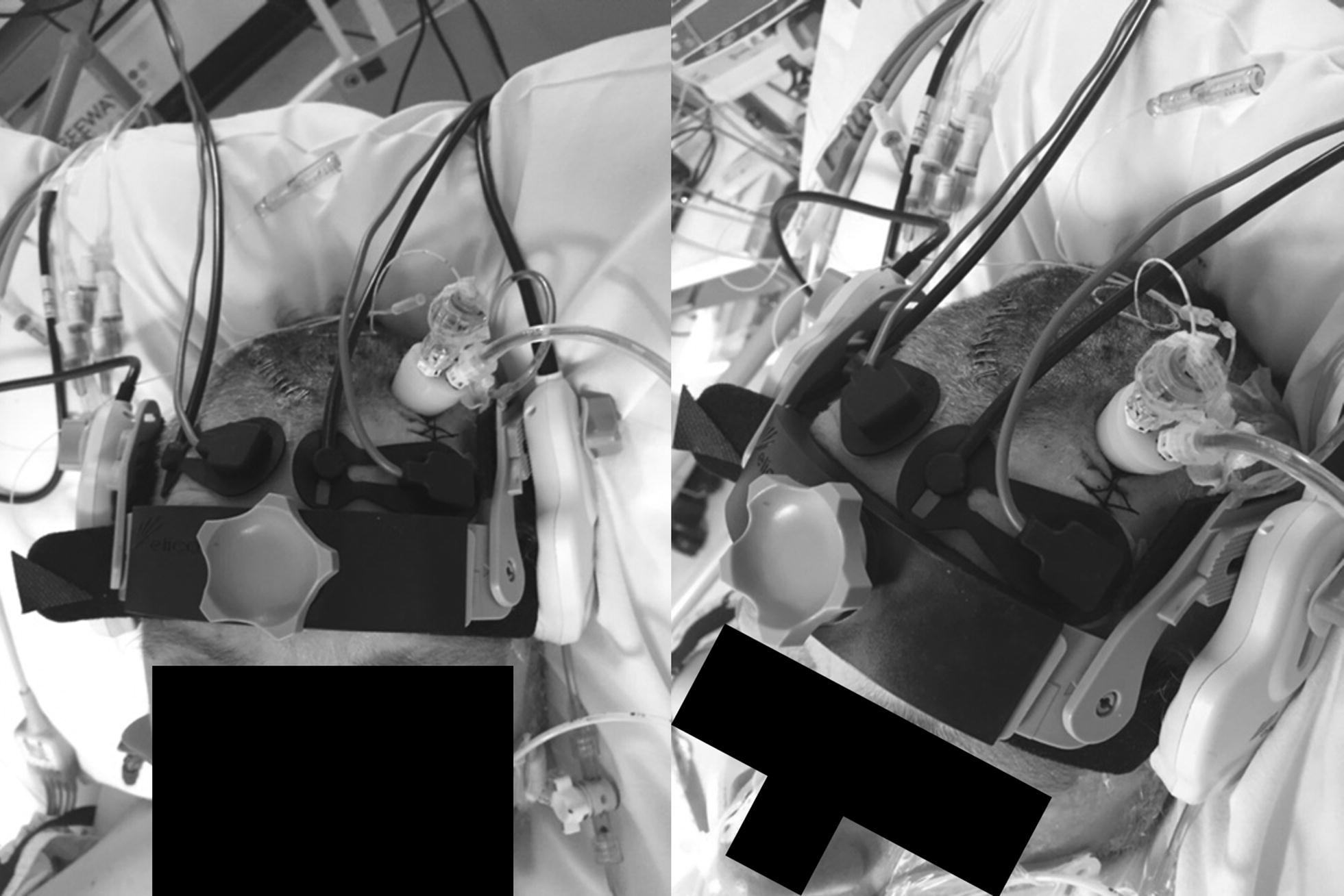
Application of Robotic TCD System in TBI patient with multi-modal monitoring. Patient has triple bolt located in left frontal area, with bifrontal NIRS pads applied (all images). Delica TCD head-band can be seen crossing the forehead, just above the orbital rims. Wheel for ratchet tightening of head-band can be seen (both upper left and right images). Robotic drive/TCD probe can be seen over transtemporal window, insonating the MCA (upper right and lower left images)

We applied the Delica EMS 9D system in moderate/severe TBI patients who were intubated, sedated and had other multi-modal monitoring in situ (i.e., both invasive and non-invasive). Our typical patient set-up can be seen in Fig. 4, including a triple-bolt (ICP, Licox, and microdialysis) with bifrontal NIRS. During the described time period, we were able to record 10 patients.

### Advantages

With above described set-up, we were able to record at least 4 h of continuous, non-interrupted, good quality TCD signals. The TCD probe held ultrasound gel for 4 h without any drying issues or need for re-application. Both the recording length and gel integrity duration are far longer than most standard TCD systems can provide. The optional rubber ring around the margins of the TCD probe appeared to also hold the gel in situ for longer. Though we must acknowledge, this rubber ring was only utilized in 2 of the 10 patients and appeared to be cumbersome at times to insonation. Thus, further testing with this added feature is required prior to definitive conclusion on its benefit being made.

Aside from gel integrity and duration of recording, we were able to capture bilateral MCA flow velocities in the presence of all other multi-modal monitoring devices described, confirming its applicability in the multi-monitoring moderate/severe TBI patients. ICM+ software ran seamlessly off of the Delica monitor, allowing for simultaneous digital signal acquisition from all multi-modal monitors.

The head-band appeared to be comfortable, with the padding protecting surgical wounds from injury, to rarely moving once secured. Once in situ, the head-band, with attached robotic drive/TCD probes tolerated patient turning in each patient, without loss of CBFV waveform or intensity. Furthermore, 2 of the 10 patients underwent portable chest x-ray while recording, with no change in signal intensity throughout this procedure. Finally, 1 of the 10 patients underwent a bedside chest-tube insertion without change in signal quality.

The robotic drive system worked well, with the search and directional functions aiding with set-up and re-acquisition of signal if it needed to be optimized. The algorithms for these two functions appeared to work well, finding the optimal angle and position of insonation. This, in the author’s opinion, was a massive “time-saver”, preventing the need to loosen the head-band or drives and manually adjust the TCD probe. Especially important in patients with numerous monitors in situ, where repeated loosening and manipulation could potentially lead to disruption of one or more of these other monitors. This advantage cannot be overstated, as the presence of automated robotic adjustment of the probe both during the initial set-up phase and during episodes of frame shift is invaluable. With this step forward in technology, the ability to obtain extended duration, mostly uninterrupted, CBFV recordings is achievable. This allows for continuous non-invasive assessment of various aspects of cerebrovascular physiology using this technology. This includes continuous measures of CBFV, pulsatility index, and cerebrovascular reactivity (in the presence of finger-cuff based continuous non-invasive blood pressure), to name a few. As robotic TCD device technology continues to improve, we are likely to see further widespread application across various pathologies, obtaining longer and higher quality recordings. This is a step towards more non-invasive means of continuous cerebral monitoring.

Finally, all plastic elements of the device were easily cleaned, allowing for rapid equipment turn-over between patients, an important aspect for those wishing to employ the device for multiple short duration recordings within a single day.

### Disadvantages

The advantages of this system far outweigh any disadvantages listed within this section. However, the system suffered from a few important initial issues/limitations, which we will highlight. Other minor annoyances with the device can be seen in the table within Additional file 2.

The main issues stemmed from initial poor real-time functioning of certain aspects of the robotic drive system. As we mentioned above, the search and direction functions appear to work beautifully. However, the two other functions (scan and track), within the initial version of the system, were less useful. The scan function, within all patients recorded, failed to select automatically the appropriate optimal position for insonation. This occurred during repeated attempts. As a result, we elected to manually position the probe using the CBFV waveform and M-mode, selecting an appropriate starting position and depth for insonation. We then applied the search and direction functions, which provided improvement and overall optimization of the signal acquired. This issue was raised with the manufacturer, whom responded promptly with a software update, leading to a remedy to the scan function. This was subsequently trialed on additional patients, confirming its functionality and fix to the previous issues.

As well, with the initial software version, the track function rarely appeared to work. This was a disappointment to us, as the hope with this function is that the drive would automatically correct for any shift in the probe during recording. There were two issues we found with this function in the initial software version. First, despite being enable, it was rarely triggered, even in the rare case when the headframe/probe shifted and lost signal. Second, when the track function was triggered, and we could audibly identify that the robotic drive was attempting to move the TCD probe, however, it never appeared to accurately correct the probe for signal loss. Both issues were likely a result of inefficiencies in the embedded algorithm for the track function. As with the scan function, our concerns were raised with the manufacturer and resolved with a subsequent, most recent version of the software. Trialing this updated software version on additional patients, the track function was correctly engaged during shift of the probe, and corrected the probe position/angle, optimizing the CBFV signal.

In addition, the overall available TCD CBFV signal recording frequency should be commented on. Currently the signal recording frequency is at 100 Hz. This is adequate for basic waveform analysis techniques. However, if one were to use this signal for more complex heart rate variability analysis, it could be argued that higher recording frequencies of 200 Hz or higher would be ideal. Thus, this might be considered a limitation of the current signal recording. However, pressure signals (as well as TCD-based CFBV) do not necessarily require as high of frequencies, and their content extends to 10–20 pulse harmonics at best. Therefore, a sampling frequency of 100 Hz may be entirely sufficient for recording those signals and this is what the majority of bedside and NCCU monitors currently offer.

Finally, we observed that in one patient with scalp soft tissue bruising, the ratcheting head-band triggered a sustained intra-cranial pressure elevation above 20 mmHg lasting 5 min, with an initial baseline ICP of 10–15 mmHg. This was promptly resolved by re-adjusting the head-band. In this one patient, however, the sedation was relatively light, and thus the ICP elevation was likely secondary to pain experienced during the head-band tightening. Nevertheless, this is an important potential complication to highlight.

### Interaction with manufacturer

An important aspect of new technologies is the responsiveness of the manufacturer to queries regarding their devices. We were fortunate enough to have this device on trial/loan for the purpose of this evaluation. As eluded above, the concerns outlined within this document were relayed to the manufacturer. They were extremely prompt in response to these raised issues. The main concerns raised above were well received and we were provided with a timely updated software version, leading to resolution of these encountered issues. Delica appears to be vigilant in providing a clinically useful TCD tool, with desires to continually improve their device, rapidly including the concerns of their clients in the process.

## Conclusions

The current Delica EMS 9D robotic TCD system provides the ability to obtain 4+ h of continuous, uninterrupted bilateral TCD recordings in critically ill TBI patients, undergoing other various invasive/non-invasive multi-modal monitoring. Further, the automated algorithms and robotic probe drive aid in TCD set-up and optimization of signal intensity during patient movements, allowing for extended duration uninterrupted recordings. This feature is a major step towards more continuous non-invasive neuromonitoring. Finally, the monitor itself provides the ability to run ICM + software, directly recording high-frequency digital signals from the robotic TCD, amongst all other multi-modal monitoring devices within moderate/severe TBI patients. We look forward to continued use of this product in the critically ill TBI population.

## Additional files

**Additional file 1.** Rubber ring construct for ultrasound gel retainment.

**Additional file 2.** Minor issues/annoyances with Delica EMS 9D robotic TCD system.

## References

[1] Kalanuria A, Nyquist PA, Armonda RA, Razumovsky A (2013). Use of transcranial Doppler (TCD) ultrasound in the neurocritical care unit. Neurosurg Clin N Am.

[2] Purkayastha S, Sorond (2012). Transcranial Doppler ultrasound: technique and application. Semin Neurol.

[3] Sorrentino E, Budohoski KP, Kasprowicz M (2011). Critical thresholds for transcranial Doppler indices of cerebral autoregulation in traumatic brain injury. Neurocrit Care.

[4] Zeiler FA, Cardim D, Donnelly J, Menon D, Czosnyka M, Smieleweski P (2017). Transcranial Doppler systolic flow Index and ICP derived cerebrovascular reactivity indices in TBI. J Neurotrauma.

[5] Zeiler FA, Donnelly J, Calviello L, Menon DK, Smielewski P, Czosnyka M (2017). Pressure autoregulation measurement techniques in adult traumatic brain injury, part I: a scoping review of intermittent/semi-intermittent methods. J Neurotrauma.

