# Supplementary figures and images for: Application of robotic transcranial Doppler for extended duration recording in moderate/severe traumatic brain injury: first experiences
Source: Crit Ultrasound J. 2018 Jul 23;10:16. doi: 10.1186/s13089-018-0097-0 (PMC6055223; doi:10.1186/s13089-018-0097-0)

**Appendix A: Rubber Ring Construct for Ultrasound Gel Retainment**


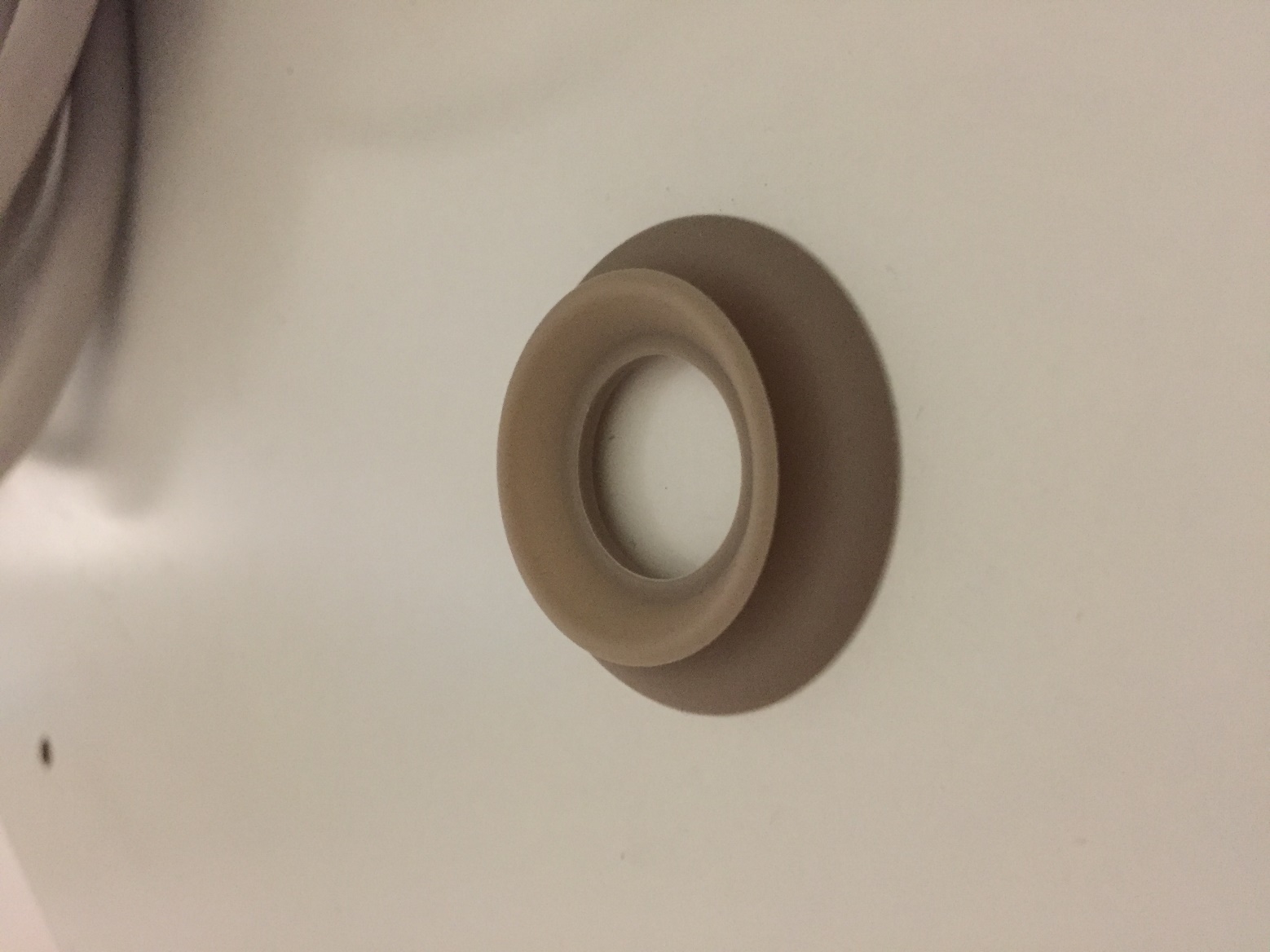


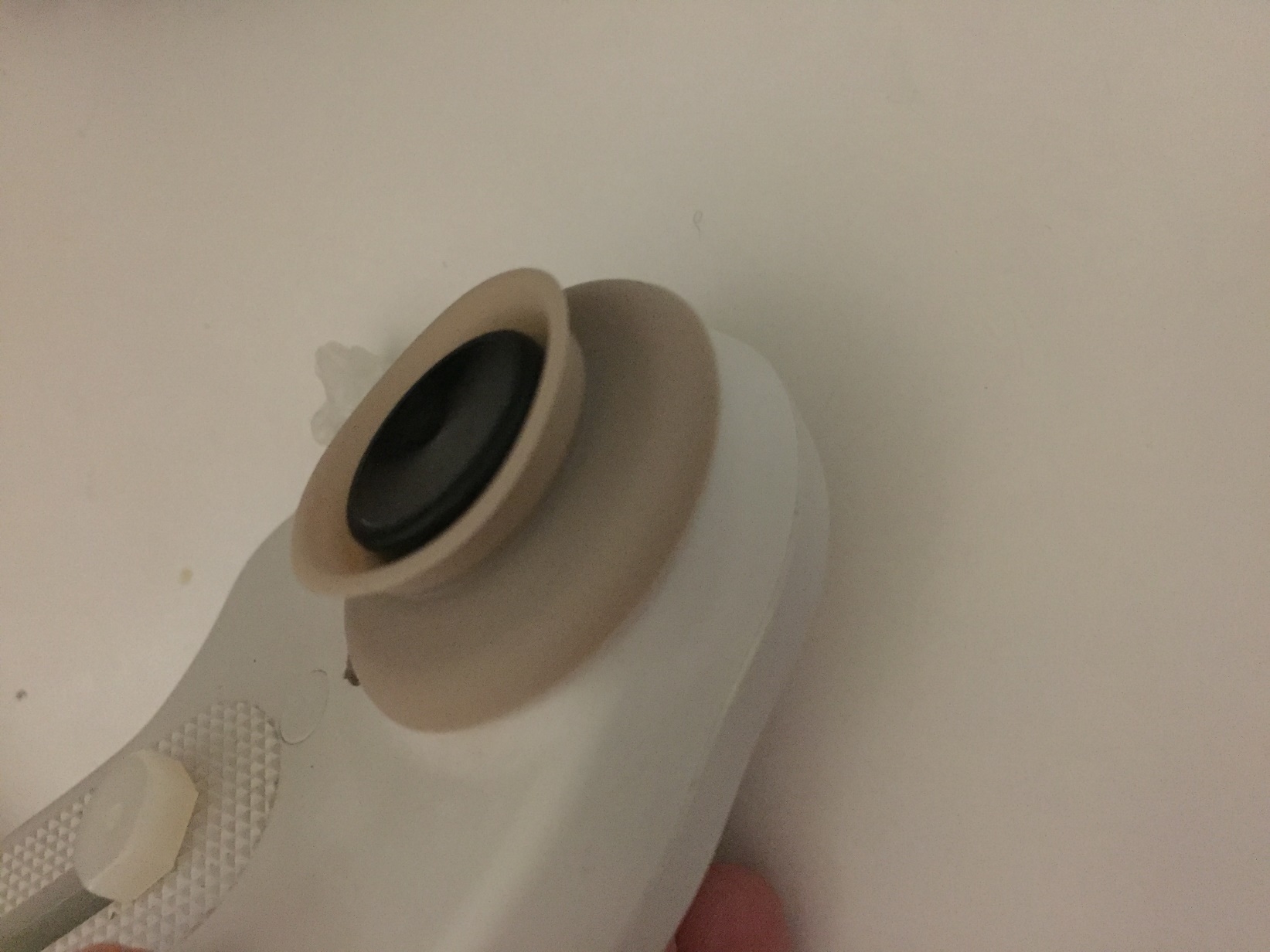

Supplement: Supplementary file 1 — Additional file 1. Rubber ring construct for ultrasound gel retainment. [file 13089_2018_97_MOESM1_ESM.docx]
