# Supplementary material for: Application of robotic transcranial Doppler for extended duration recording in moderate/severe traumatic brain injury: first experiences
Source: Crit Ultrasound J. 2018 Jul 23;10:16. doi: 10.1186/s13089-018-0097-0 (PMC6055223; doi:10.1186/s13089-018-0097-0)
Supplement: Supplementary file 2 — Additional file 2. Minor issues/annoyances with Delica EMS 9D robotic TCD system. [file 13089_2018_97_MOESM2_ESM.docx]

Appendix B: Minor Issues/Annoyances with Delica EMS 9D Robotic TCD System

| **Minor Issues** |
| --- |
| 1. *Set-up Time* – Intubated, sedated TBI patients with multi-modal monitoring, it takes ~30 minutes with one operator to set everything up safely, given the head-band must be disassembled and reassembled around the patient’s head, ensuring other invasive cranial monitoring, central access and endotracheal tube are not disturbed. |
| 1. *C-collar –* Given some manipulation of the patient’s head to secure the device in these complex patients, authors were uncomfortable placing in those with C-collar and uncertain stability status of the cervical spine. |
| 1. *Decompressive Craniectomy* – Given the probe holder is a head-band, secured circumferentially, this cannot be safely applied in those patients with a craniectomy. |
| 1. *Headband Thickness* – Head-band is ~1-1.5 inches thick, leaving only a small area to apply bifrontal NIRS pads in the presence of a triple bolt. |
| 1. *Neoprene Pads -*  Though exchangeable, the pads take time to clean properly with disinfectant |
| 1. *Headband Size* – Despite being readily adjustable, we’ve found patients with small stature may have head circumferences that are too small for the manual straps and ratcheting system to tighten effectively. |

C-collar = cervical collar, NIRS = near infrared spectroscopy.
